# The Complement System in the Pathophysiology of Pregnancy and in Systemic Autoimmune Rheumatic Diseases During Pregnancy

**DOI:** 10.3389/fimmu.2020.02084

**Published:** 2020-08-27

**Authors:** Cecilia Beatrice Chighizola, Paola Adele Lonati, Laura Trespidi, Pier Luigi Meroni, Francesco Tedesco

**Affiliations:** ^1^Experimental Laboratory of Immunological and Rheumatologic Researches, Istituto Auxologico Italiano, IRCCS, Milan, Italy; ^2^Department of Obstetrics and Gynaecology, Fondazione Cà Granda, Ospedale Maggiore Policlinico, Milan, Italy

**Keywords:** complement, pregnancy, obstetric complications, anti-phospholipid syndrome, systemic lupus erythematosus

## Abstract

The complement system plays a double role in pregnancy exerting both protective and damaging effects at placental level. Complement activation at fetal-maternal interface participates in protection against infectious agents and helps remove apoptotic and necrotic cells. Locally synthesized C1q contributes to the physiologic vascular remodeling of spiral arteries characterized by loss of smooth muscle cells and transformation into large dilated vessels. Complement activation triggered by the inflammatory process induced by embryo implantation can damage trophoblast and other decidual cells that may lead to pregnancy complications if the cells are not protected by the complement regulators CD55, CD46, and CD59 expressed on cell surface. However, uncontrolled complement activation induces placental alterations resulting in adverse pregnancy outcomes. This may occur in pathological conditions characterized by placental localization of complement fixing antibodies directed against beta2-glycoprotein 1, as in patients with anti-phospholipid syndrome, or circulating immune complexes deposited in placenta, as in patients with systemic lupus erythematosus. In other diseases, such as preeclampsia, the mechanism of complement activation responsible for complement deposits in placenta is unclear. Conflicting results have been reported on the relevance of complement assays as diagnostic and prognostic tools to assess complement involvement in pregnant patients with these disorders.

## Introduction

Motherhood has become a feasible option in recent years even for women with rheumatic diseases, thanks to the marked improvement in the diagnostic modalities and therapeutic approaches developed in the field of rheumatology. Clinicians devoted to the management of pregnant women with systemic autoimmune rheumatic diseases have accumulated a particular experience in systemic lupus erythematosus (SLE), which disproportionately affects women during childbearing age (1, 2). A unique scenario in the obstetric/rheumatologic field is provided by anti-phospholipid syndrome (APS) that manifests with pregnancy complications and vascular thrombosis. Anti-phospholipid antibodies (aPL) interfere directly with pregnancy progression as documented by the ability of aPL administered to pregnant animals to reproduce the disease, offering an invaluable tool to investigate the pathogenic mechanisms implicated in obstetric complications (3).

Pregnancy has become a relatively frequent condition in SLE and APS women over the last decade and its incidence in patients with these diseases does not appear to be different from that of normal pregnant women. However, despite the progress made in recent years, pregnancies in these conditions are still burdened by a high rate of obstetric complications, mainly in terms of pre-eclampsia, preterm delivery, and intrauterine growth restriction (IUGR) (4) and a tight control is recommended for a positive pregnancy outcome (4, 5). Thus, surrogate biomarkers are highly needed in early gestation to identify women at risk of adverse pregnancy outcome and to monitor progression thanks to serial sampling. Similarly, a better elucidation of the pathogenic steps could lead to the development of more effective targeted therapeutic strategies. In this regard, the complement (C) system has attracted much attention as candidate pathogenic effector of autoimmune and non-autoimmune pregnancy complications and surrogate biomarker to stratify obstetric risk in the general population of pregnant women. Earlier notions on C levels refer to lupus pregnancies, which has become a topic of particular interest following the observation of an association between serum C3 and C4 levels and disease flares in non-gravid patients. *In vivo* APS models have progressively unraveled the importance of C in the pathogenesis of obstetric complications. It is important to emphasize that C is a complex system with a subtle balance between protective and damaging effects. This balance undergoes physiologic modifications during gestation, which may bias the accuracy of results. It is thus timely to review available evidence on the actual and potential relevance of C as pathogenic effector of pregnancy complications and biomarker of obstetric outcome in women with systemic autoimmune rheumatic conditions.

## The Complement System: A Double-Edged Sword

**FIGURE 1:**
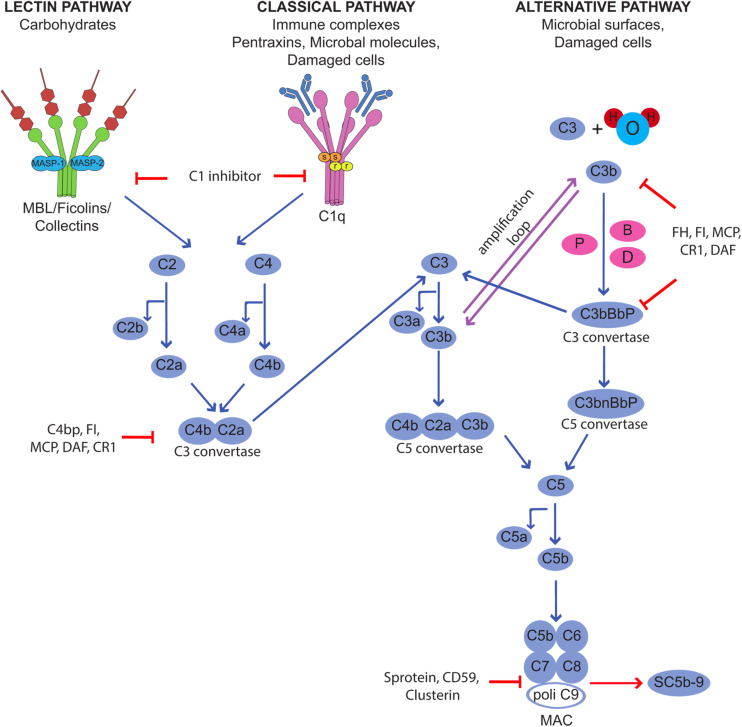
Activation of the complement cascade through the classical, lectin, and alternative pathways and its control by regulators and inhibitors.

Complement is a humoral component of the innate immune system that contributes to host defense neutralizing infectious agents, removing immune complexes and clearing apoptotic and necrotic cells. The protective function is accomplished through the action of biologically active products that are released as a result of C activation and exert their effects by enhancing phagocytosis, causing cell cytotoxicity, and promoting inflammation (6). Furthermore, the C system plays an important role in bridging innate and adaptive immunity, as its activation is critical for the development of adaptive immunity (7, 8). C is a versatile system organized to provide protection from a variety of targets using different recognition molecules that sense danger signal coming from foreign agents and altered self and trigger the classical, lectin and alternative activation pathways (9) (Figure 1). All pathways converge at the level of C3 and proceed along a common terminal pathway leading to the release of the anaphylotoxins C3a and C5a, cell deposition of C3b and assembly of the terminal C complex. The complex inserts into the cell membrane as membrane attack complex (MAC) forming membrane pores that are responsible for cell lysis. Alternatively, the complex that fails to exert a cytotoxic effect accumulates in blood and extravascular fluids as soluble SC5b-9, which can trigger cytokine synthesis, stimulates inflammation, and induces vascular leakage (10, 11).

Although C is quite selective in focusing the defense activity on dangerous targets recognized by the initiators of the activation pathways, the effector molecules released during the activation process are unable to discriminate between self and non-self and may easily bind to bystander cells. This may happen in physiological conditions, as the C system usually operates at a steady state level of activation, and the split product C3b continuously formed in the circulation and in the extravascular fluid is deposited on the cell surface. As a result, normal cells and tissues are exposed to C attack that may be destructive under conditions of unrestricted C activation. Fortunately, the potential danger that may derive from an undesired C attack is prevented by the protective effect of C regulators and inhibitors present in the fluid phase and widely expressed also on the cell surface (12). These molecules act at various steps of the C sequence and control the function of the system in various ways preventing the assembly of C complexes, favoring their disassembly, and neutralizing the activity of the biologically active products. The membrane−bound regulatory proteins CD46, CD55, and CD59 play a particularly important role in cell protection and may be used by microorganisms and cancer cells to evade C attack. They are often present on the same cells and combine their efforts to control critical steps of C activation at the level of C3 convertases (CD55 and CD46) and MAC assembly (CD59).

The exquisite selectivity of the C system for dangerous targets can be circumvented by C-fixing autoantibodies that react with self-antigens expressed on normal cells and tissues and triggers C activation leading to cell death and tissue damage. However, it is important to emphasize that C activation does not necessarily result in tissue injury, but it may also have beneficial effect contributing for instance to promote angiogenesis and wound healing (13) and also to eliminate inappropriate synaptic connections during development (14).

The role played by the C system in several clinical conditions can now be easily evaluated by functional analysis of the three pathways of C activation and the measurement of activation products recognized by antibodies directed against neoepitopes expressed on cleaved proteins.

## The Growing Importance of Complement in Healthy Pregnancy

**FIGURE 2:**
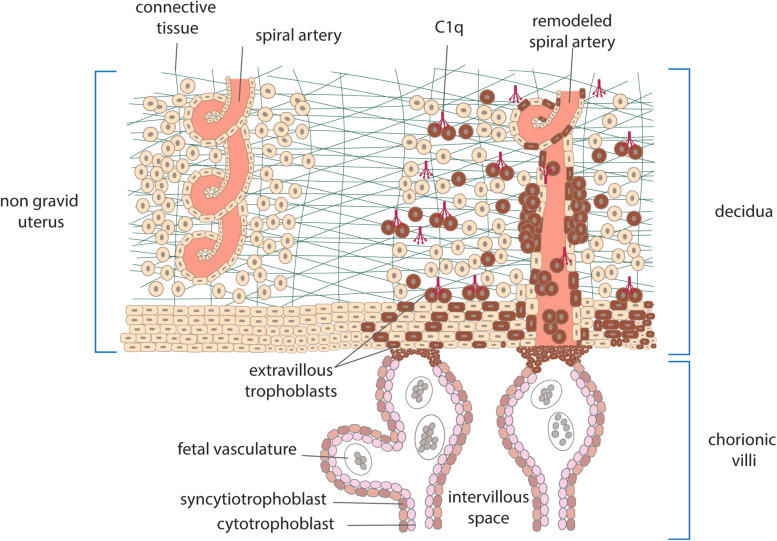
Schematic representation of human placenta showing anchoring villi attached to maternal decidua. Extravillous trophoblasts depart from the villi and invade the decidua surrounding the spiral arteries. Other cells enter the lumen of the arteries as endovasculat trophoblasts and partially replace lining endothelial cells. C1q produced by trophoblasts is used to promote their migration and interaction with endothelial cells.

Embryo implantation is a real challenge for the maternal immune system which is confronted with paternal antigens expressed on the embryo and the fetus and yet does not mount an immune response leading to its rejection, as it would happen with incompatible organ transplants. Both the trophoblasts that cover the villi bathed into maternal blood and the extravillous trophoblasts invading the maternal decidua represent the main source of these antigens. Villous trophoblasts form a physical double-layer barrier between the fetus and the mother and serve the important function to protect the fetus from maternal immune attack allowing only selective passage of nutrients and defense factors from the mother. Conversely, the extravillous trophoblasts depart from the anchoring villi attached to maternal decidua and contribute to tissue remodeling required for successful implantation. Besides the important role in local defense against infectious agents that may damage the fetus, C has attracted particular attention in recent years for the involvement in the physiologic changes that occur in placenta. The system is present in the maternal blood that circulates in the intervillous space and may be activated by cell-debris of trophoblasts and possibly immune complexes that have been detected in healthy pregnancy (15). Higher levels of MBL, C4, and C3 and of the activation products C4d, C3a, and SC5b-9 have been reported in pregnant women compared to non-pregnant controls (16), while the circulating levels of C1q do not fluctuate and remain relatively stable throughout normal pregnancy (17, 18). C activation in maternal blood represents a continuous risk for villous trophoblasts and may cause cell damage and impairment of the barrier integrity. This dangerous situation is kept under control by the expression of C regulatory proteins on trophoblast surface including CD55, CD46, and CD59, that act at different steps of the C sequence promoting the decay of the C3 convertases, favoring the inactivation of C3b and C4b and preventing the assembly of C5b-9 (19, 20). C components are also synthesized by different types of cells present in decidua including macrophages, trophoblasts and endothelial cells (21) and form a local system that may operate as a local defense system. Embryo implantation in maternal uterus is associated with an inflammatory-like process induced by proteolytic enzymes that are released by extravillous trophoblast invading the decidua (21). The extensive tissue remodeling caused by trophoblast invasion leads to local recruitment of natural killer cells (NK) and other cells of the innate immune system and activation of the C system, which has limited damaging effect due to the widespread distribution of C regulators. Data collected in recent years have revealed an important role of C1q in the physiological remodeling of decidual spiral artery characterized by partial replacement of endothelial cells by endovascular trophoblasts that migrate upward from the anchoring villi. C1q is synthesized and expressed on the cell surface of both decidual endothelial cells lining the inner side of the spiral arteries and endovascular trophoblasts and serve the important function to establish a molecular bridge between the two cell types (22) (Figure 2). The C1q-mediated cellular crosstalk leads to the formation of mosaic vessels with an inner layer formed by the mixture of endothelial cells and trophoblasts. C1q is also synthesized and secreted by the extravillous trophoblasts as soon as they start moving away from the anchoring villi and is required for trophoblast invasion of the decidua (Figure 2). By binding to the extracellular matrix, C1q promotes the adhesion and the migration of extravillous trophoblasts that reach the spiral arteries forming cuffs and contribute to the vascular remodeling (23).

## Complement and Adverse Pregnancy Outcome

Evidence collected over the years has revealed that C plays a dual role during pregnancy. On one hand, the system promotes the physiologic changes at fetal-maternal interface required for a successful pregnancy, and on the other hand it may cause placental damage leading to impairment of the regular progression of gestation. C abnormalities have been reported in several obstetric complications including early pregnancy loss, pre-term birth, and pre-eclampsia. The relevance of C in recurrent miscarriages is supported by data obtained from animal models suggesting a key role for C5a. C activation and interaction of this anaphylatoxin with C5a receptor have been shown to induce the release of soluble vascular endothelial growth factor receptor ultimately resulting in impaired angiogenesis and adverse pregnancy outcomes (24, 25). A role in mediating abortion has also been advocated for anti-C1q antibodies as suggested by the findings that these antibodies administered to pregnant animals induce fetal loss, and that both the prevalence and the titre of anti-C1q antibodies are significantly higher in women with unexplained recurrent pregnancy loss than in healthy parous women (26). Conflicting data have been reported on the circulating levels of C components and C activity in women with early pregnancy loss. While increased levels of C3 and C4 were found in one study and proposed as predictor of fetal loss (27), hypocomplementemia was documented in another study in women with recurrent miscarriages (28). Interestingly, a significant decrease in the placental expression of C regulators CD46 and CD55 associated with excessive C activation has been observed after spontaneous abortion, reaffirming the importance of inhibiting C activation to ensure a successful pregnancy (29). Local C activation is supported by the finding of C4d deposits documented by immunohistochemical analysis of the placentae of women with recurrent miscarriages (25). It has been estimated that approximately 20% of otherwise unexplained early pregnancy loss are due to hypocomplementemia (25).

A wealth of data has been collected over several years on the involvement of C in pre-eclampsia, a disorder of pregnancy that affects 3 to 5% of women in the late phase of pregnancy and is characterized by hypertension and proteinuria. Dysregulated angiogenesis is believed to be implicated in the pathogenesis of the disease, as documented by elevated circulating levels of soluble vascular endothelial growth factor receptor 1 (sFlt-1) (30). Animal models have shown that C1q-deficient pregnant mice manifest the key features of human disease such as hypertension, albuminuria, endothelial dysfunction, decreased placental vascular endothelial growth factor, and elevated levels of sFlt-1 providing convincing evidence that C1q protects against pre-eclampsia (31). Consistent with this *in vivo* observation, Agostinis, and colleagues (17) published data indicating that the serum levels of C1q was markedly decreased in both early and late onset forms of pre-eclampsia. Likewise, women with early onset pre-eclampsia are twice as likely to carry deficiency in C4A or C4B suggesting that C4 may also contribute to prevent the onset of pre-eclampsia (32). The reduced concentration of C1q observed in patients with overt pre-eclampsia cannot be used as predictive marker of the disease because the analysis of serum samples collected at an early phase of pregnancy from women who later developed preeclampsia failed to show a decrease in C1q level (17). Mannose binding lectin (MBL) seems to have an opposite effect to that of C1q since the level is elevated in patients with severe pre-eclampsia (33, 34). Furthermore, MBL strongly inhibits the interaction of extravillous trophoblast with C1q and interferes with the process of cell migration (34), suggesting the contribution of MBL to the pathogenesis of the disease.

It has been postulated that C activation following placental ischemia may induce hypertension and impair fetal growth via the endothelin pathway (35). Analysis of C activation products in patients with pre-eclampsia has revealed increased serum levels of C3a, C5a, and SC5b-9 (16, 36, 37) and C activation products have also been detected in the urine of patients with a severe form of the disease as a result of C-mediated renal injury (38). High levels of the activation product of the alternative pathway Bb have been observed in the early phase of pregnancy in women who later developed pre-eclampsia and proposed as an early biomarker of this disease. The finding of high mRNA expression of the membrane C regulators CD55 and CD59 in placenta specimens from pre-eclamptic women has been interpreted as a compensatory attempt to limit local C activation (32). C4d is the C split product most frequently seen in pre-eclamptic placentae, particularly on syncytiotrophoblast, with focal or diffuse staining patterns (39), and the degree of C4d and MAC deposition in the placental tissue is strongly correlated with sFlt1 levels in pre-eclamptic patients (40). Available evidence suggests that activation of the C system is involved in spontaneous preterm birth. Lynch and coworkers (41) measured the circulating levels of Bb, a marker of alternative pathway activation, in pregnant women in the early phase of gestation and found that those with elevated levels were more likely to experience preterm delivery. They propose Bb as a predictor of this adverse pregnancy outcome that develops in late gestation before 34 weeks. An essentially similar conclusion was reached measuring the levels of C3a under the same experimental conditions and again higher levels were associated with preterm birth (42). The increased levels of C5a observed in women with preterm delivery suggest that C5a, by reacting with C5aR, plays a role in the pathogenesis of preterm labor (43).

## Complement and Obstetric Anti-Phospholipid Syndrome

**FIGURE 3:**
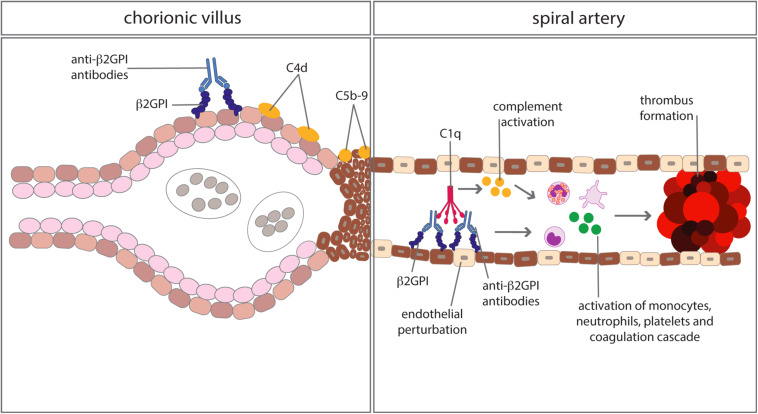
Complement-mediated biological effects at placental level in anti-phospholipid syndrome. Complement activated by antibodies interacting with β2GPI bound to trophoblasts and vascular endothelium of decidual vessels leads to cell damage and promotion of thrombus formation.

Anti-phospholipid syndrome is an acquired prothrombotic condition characterized by vascular occlusive events occurring in vessels of different size and/or obstetric complications. Adverse pregnancy outcomes include three or more spontaneous abortions before 10 weeks of gestation, one or more unexplained fetal death at or beyond week 10 of gestation, one or more preterm delivery before 34 weeks due to severe pre-eclampsia, HELLP syndrome (hemolytic anemia, elevated liver enzymes, low platelet count) or placental insufficiency. aPL are the serum biomarkers of APS, routinely detected by a functional assay, named lupus anticoagulant (LA), and two solid phase assays identifying IgG and IgM antibodies against cardiolipin (aCL) and beta2-glycoprotein I (anti-β2GPI). β2GPI, the main antigenic target of aPL, is a five domain (D) glycoprotein comprising four C control protein (CCP)-like domains (DI-DIV) and one domain (44) with a large lysine loop which allows β2GPI to interact with anionic phospholipids and other molecules on cell surfaces, coagulation factors, platelets, and complement (45). Antibodies against β2GPI co-localize with their target antigen on trophoblasts and decidual endothelial cells in immunized animals that had received fluorescein-labeled β2GPI (46) and interfere with pregnancy progression by impairing the function of the developing placenta. The antibodies exert their effect on the maternal side, promoting a negative imbalance of angiogenic factors that inhibits endometrial angiogenesis. Furthermore, they act on trophoblasts inducing apoptosis and inhibiting the secretion of β human chorionic gonadotropin and matrix metalloproteinases (MMP) required for invasion of decidua, and in complex with β2GPI activate the classical pathway of the C cascade (47). Several clinical studies have examined the activation of the C system in pregnant patients with APS and its contribution to pregnancy complications. Decreased serum levels of C4 and C3 have been reported in approximately one third of patients with APS (48) and the follow-up of these patients throughout pregnancy revealed that the C4 and C3 levels remained persistently low compared to the values of control pregnant women when normalized for the trimester of gestation (49). As shown in Table 1, lower levels of C3 and C4 were found to correlate with adverse obstetric outcomes in some studies (50, 51), but not in others (49, 52). Data obtained from a prospective study of APS pregnant women led De Carolis et al. (51) to suggest that reduced C3 and C4 levels should be regarded as predictors of lower neonatal birth weight and preterm delivery. A multicenter study performed in Japan showed that low levels of C3 and C4 represent a risk factor for hypertensive disorders of pregnancy (52). Evaluation of biologically active circulating products of the C system in APS pregnant patients offers more direct insights on C activation in this clinical condition. Blood samples from 161 aPL positive women including 60 with SLE were analyzed for the presence of Bb and SC5b-9 and increased levels of both activation products were found in all patients with adverse obstetric outcome (53). This finding has been confirmed by a more recent study, reporting higher C5a and C5b-9 levels in APS pregnant patients with pregnancy complications compared to healthy pregnant women (54). More convincing evidence supporting the role of C in inducing aPL-dependent placental damage and pregnancy failure has been obtained from the immunohistochemical analysis of placental tissue for C deposits. The presence of C4d in placentae from APS women has been documented at the fetal-maternal interface, in particular on syncytiotrophoblast basement membrane, and to some extent also on extravillous trophoblasts of the basal plate by three groups (55–57). C4 deposits were found to be associated with intrauterine fetal death (55) and placental abnormalities including decidual vasculopathy, increased syncytial knots, and villous infarcts (57). Two groups have documented deposits of C5b-9 mainly localized on extravillous trophoblasts of placentae from aPL-positive women and observed no difference in the staining intensity between APS and control groups (56, 57). These findings are in contrast with the data obtained by Scambi et al. who reported higher levels of C5b-9 solubilized from APS placentae compared to controls, in particular in APS patients who experienced a pregnancy complication (54). Our group has conducted a prospective study on 13 APS patients with medium to high titers of anti-β2GPI antibodies and positive LA who had pregnancies that resulted in one abortion, four fetal losses, and eight preterm deliveries (58). Histological and immunohistochemical analysis revealed placental abnormalities characterized by decidual vasculopathy and intervillous thrombi and deposition of IgG, IgM, C1q, C4, and C3 suggesting C activation through the classical pathway. Interestingly, C5b-9 was detected in all placentae and was localized on the surface of syncytiotrophoblasts, intervillous fibrin and decidual vessels supporting its contribution to tissue damage. Taken together, these findings suggest that complement activation is involved in placental pathology acting both on villous trophoblast and on endothelial cells of decidual vessels (Figure 3). Animal models of APS developed by infusing patient’s IgG have provided key information on the role played by the C system in eliciting placental abnormalities and adverse pregnancy outcomes. Mice deficient in C3, C4, C5, and factor B were found to be resistant to aPL-induced fetal loss (59–61) and similar results were obtained using a C3 convertase inhibitor, a C5a receptor antagonist or anti-C5 antibodies (60). C5a has been identified as the main mediator of fetal injury by interacting with C5aR expressed on polymorphonuclear leukocytes and by stimulating the release of tissue factor and tumour necrosis factor (TNF)-α, which in turn promotes inflammation (62, 63). Given the growing evidence implicating C activation as key contributor to the pathogenesis of the clinical manifestations of APS, C inhibitors have been considered good candidates for the therapy of APS. Although the neutralizing anti-C5 antibody eculizumab has been used successfully in treating patients with catastrophic APS and in preventing re-thrombosis in patients undergoing surgical intervention (58, 64), very few information is available on its use in APS pregnant patients except for an anecdotal report of a patient who received eculizumab to prevent severe pregnancy complications (65). A non-C fixing anti-β2GPI monoclonal antibody that was shown to prevent fetal loss in aPL-treated pregnant mice offers an alternative therapeutic approach (66). The advantage of this antibody is to target the β2GPI protein constitutively expressed on villous and extravillous trophoblasts as well as on the endothelium of decidual vessels with relatively high affinity and to compete with antibodies from APS patients.

**TABLE 1:** Studies assessing the correlation of C3 and C4 serum levels with obstetric outcome in pregnant women with anti-phospholipid syndrome.

Author, year	Number of APS patients/pregnancies	Study design	Timing of C testing	Control group	Main findings
Ruffatti, 2011 (50)	114/114 All PAPS	Retrospective	Baseline and at the end of pregnancies	None	- Hypocomplementemia was associated with adverse pregnancy outcome at univariate analysis.
De Carolis, 2012 (51)	47/47 PAPS/SAPS (No SLE)	Prospective	Within 20 gestational weeks	None	- Hypocomplementemia was associated with fetal loss and preterm delivery at univariate analysis. - Women with hypocomplementemia had lower neonatal birth weight. - Hypocomplementemia was not associated with PE and IUGR at univariate analysis.
Reggia, 2012 (49)	45/57 PAPS	Retrospective	I-II-III trimester	49 women with UCTD/SjS 175 healthy pregnant women	- Hypocomplementemia was not associated with adverse pregnancy outcome in PAPS women. - Women with PAPS had lower C3 and C4 than healthy women, but similar to UCTD and SjS.
Deguchi, 2017 (52)	69/81 PAPS/SAPS (mainly SLE)	Retrospective	NS	None	- Hypocomplementemia was not associated with pregnancy loss, premature delivery and IUGR. - Hypocomplementemia was associated with hypertension at multivariate analysis.

*PAPS, primary anti-phospholipid syndrome; SAPS, secondary anti-phospholipid syndrome; C, complement; NS, not specified; SLE, systemic lupus erythematosus; UCTD, undifferentiated connective tissue disease; SjS, Sjögren syndrome; PE, pre-eclampsia; and IUGR, intra-uterine growth restriction.*

## Complement and Systemic Lupus Erythematosus in Pregnancy

Systemic lupus erythematosus is a complex multisystem autoimmune disease with a highly heterogeneous presentation ranging from laboratory abnormalities to multi-organ inflammation and failure and characterized by the production of autoantibodies directed against double stranded DNA and several other autoantigens. The clinical manifestations of SLE are underpinned by several etiopathogenic mechanisms, such as deregulated production of autoantibodies against cellular constituents, abnormal cytokine release, innate, and adaptive immune alterations. Impaired clearance of apoptotic debris is believed to mediate sustained antigen presentation to B cells, ultimately resulting in exaggerated autoantibody production (67). C activated by immune complexes formed in the circulation and at tissue level following interaction of autoantibodies with their target antigen is involved in the pathogenesis of the clinical manifestations of SLE. Some of these autoantibodies are directed against C components, as is the case of anti-C1q antibodies detected in nearly one third of patients with lupus nephritis and thought to have important pathogenic effects in the development of the disease (68). Immune complexes containing anti-C1q antibodies were found to be more potent C activators than classical immune complexes (69). Deposits of C components and C activation products including the terminal complex have been documented in the kidney of SLE patients co-localized with immune complexes suggesting the involvement of C in tissue damage (70). Data accumulated over several years have shown that C plays a paradoxical role in SLE. On the one hand, C activated by immune complexes stimulates inflammation, and causes tissue lesions. The important role played by C is suggested by the beneficial effect of eculizumab in patients with severe lupus nephritis resistant to conventional therapy (71, 72). Further evidence supporting the contribution of C activation to SLE pathogenesis is provided by lupus-prone mouse models, such as NZB/W and MRL/lpr mice, that share with the human disease similar features including autoantibodies production, hypocomplementemia, circulating and glomerular-bound immune complexes, and severe nephritis (73). Treatment of these mice with anti-C5 monoclonal antibodies resulted in improvement of nephritis, reduced proteinuria and prolonged survival (74). On the other hand, C deficiency is now recognized to be a risk factor for SLE development, based on the finding that genetic deficiencies of the early components of the classical pathway from C1q to C4 are associated with the onset of SLE (75). C1q-deficient individuals have the highest susceptibility to SLE due to the role of C1q in the removal of apoptotic cells. The disease occurs in up to 55–75% of individuals with this genetic defect and presents with characteristic clinical features including early age of onset, no gender predilection, low frequency of anti-dsDNA antibodies, prominent photosensitivity, and fewer renal symptoms (76). Given this background, it is not surprising that the C system has attracted particular attention as an important marker of disease activity in SLE patients. It’s long been known that C activation in SLE is accompanied by a secondary reduction in circulating C levels and increase in C split products. Importantly, the decrease in C1q, C3, and C4 levels correlates with disease activity and precedes clinically evident flare (77), even though the decrease in C levels are not invariably associated with disease flares (78). Despite the technical and biological limitations, measurements of C3 and C4 have been included not only in the recent classification criteria for SLE, but also in the disease activity indexes such as SLEDAI (79). Recently, C deposition on immune cells was proposed as a more robust method to diagnose and monitor SLE and a panel of parameters including C4d-deposition on B cells and erythrocytes was suggested (80, 81).

Pregnancies in patients with SLE have always been regarded to be at risk, even though the rates of fetal loss and maternal mortality have steadily decreased over the years (82). However, pregnant women with SLE still display an increased hazard of premature delivery and IUGR irrespectively of disease activity, while the odds for pre-eclampsia is elevated in women with active disease only (83). Hypocomplementemia during gestation has been identified as one of the multiple predictors of poor pregnancy outcome in SLE pregnancies including high disease activity in the 6 months before conception, use of anti-hypertensive medications, non-white ethnicity, aPL positivity, and a history of nephritis or active nephritis at conception. Data on C levels in SLE pregnant women vary considerably in different studies, depending on the composition of the study cohort. Thus, hypocomplementemia is prevalent in certain disease manifestations, such as lupus nephritis. C4 level is a more reliable marker of renal involvement in SLE since low C4 at baseline and a history of previous kidney disease have been found to be independently associated with a higher risk of developing active nephritis in pregnancy (84). Pregnant women with lupus nephritis display significantly lower levels of C3 and C4 more often than other SLE subjects (85). Data on the association of C levels with poor pregnancy outcome can be obtained from studies that assess several clinical and laboratory variables of SLE as predictors of adverse obstetric outcomes, but there is no universal agreement on the clinical significance of complement levels as biomarkers in lupus pregnancies. Indeed, few authors deny a predictive role for C3 and C4 whereas in other studies low C3 and/or C4 levels have been associated with adverse pregnancy outcomes such as spontaneous abortion, premature birth and stillbirth (86–88). Unfortunately, a control group of healthy pregnant women was not included in these studies, and the C levels were not normalized for the gestational age, thus limiting the conclusions on the relationship between C3 and C4 levels and pregnancy outcomes. Changes in C levels were evaluated in 386 SLE patients throughout gestation in the PROMISSE study, and a smaller increase in C3 levels in the second and third trimesters was observed in women with adverse obstetric outcome compared to women with uneventful pregnancy, though the difference was not significant (87). Because of the well-known fluctuation of C levels throughout pregnancy, it is not surprising that low *C* values may not correlate with disease activity in pregnant patients with SLE except when they are lower than those expected in normal pregnant women at the same gestational age (89, 90). The C activation products Ba, Bb and SC5b-9 represent more sensitive indicators of C activation and may be useful to predict and diagnose flares in pregnant SLE patients (89). In the PROMISSE cohort, these markers of C activation (Bb and SC5b-9) were detected in the circulation in early gestation among those SLE/aPL + women who later developed pregnancy complications (53). Analysis of placentae from aPL-negative SLE patients by Matrai and colleagues revealed signs of tissue malperfusion, infarction and intervillous thrombi and increased deposits of C4d and C5b-9 on syncytiotrophoblasts and extravillous trophoblasts compared to controls (56). The extent of C4d deposition was found to be inversely correlated with low placental and birth weight (91).

## Conclusion

Complement is a versatile system that shows exquisite adaptation to environmental changes and is able to recognize dangerous exogenous and endogenous agents and structures. Besides exerting protective functions, C is now recognized to promote functions unrelated to host defense including tissue repair and remodeling. Data collected in recent years have shown that C plays an important role in the structural organization of placenta at fetal-maternal interface contributing to vascular remodeling of spiral arteries in maternal decidua, a critical process required for the regular progression of pregnancy. However, placenta is not exempt from potential damage that may derive from activation products released as a result of general or local C activation leading to adverse pregnancy outcomes. C exerts a direct damaging effect in clinical situation such as APS as suggested by the failure of antibodies to induce fetal loss in C-deficient animals or treated with neutralizing antibodies to C components. The finding of C components at placental level both in APS patients and in animal models further supports the involvement of C in the onset of placental alterations and has both diagnostic and therapeutic implications. Measurement of C levels is routinely performed in many obstetrics/rheumatology joint clinics to monitor APS pregnancies, but hypocomplementemia does not seem to be a reliable marker to predict pregnancy loss in these patients. More sophisticated and sensitive methods have been proposed to monitor C activation, as is the case of cell-bound C split products. The recent report of a higher percentage of C4-positive B lymphocytes, erythrocytes, and platelets in patients with obstetric and thrombotic manifestations compared to controls (92) suggests that this assay may be an interesting tool to explore C activation in pregnant women with APS or SLE. Preventive treatment with neutralizing antibodies or other reagents aimed at controlling C activation is a promising therapeutic approach in APS. Indeed, heparin currently used as treatment of choice for pregnant APS women together with low dose aspirin was shown to inhibit C activation and to prevent cell binding of β2GPI as a result of interaction with the heparin-binding site located on DV (93). C is most likely involved in the adverse pregnancy outcome observed in patients with SLE, a prototypical C-mediated disease. Currently, measurement of C levels is requested by the obstetricians to differentiate between nephritis and pre-eclampsia in SLE patient with proteinuria. C3 and C4 levels normally rise in patients with pre-eclampsia, while drops in C3 and C4 levels, coupled with a rising anti-dsDNA titre, are more likely associated with disease flares (94). However, despite many studies conducted to identify predictors of adverse outcome in lupus pregnancies, there are no clear data supporting the association between fluctuation of C levels and disease flare during gestation. Conclusive data on C-mediated tissue damage associated with adverse pregnancy outcomes can be provided by the histological analysis of placenta samples from patients. To be informative, the results should be compared with those of healthy controls of the same gestational age as the phenotype changes with the progression of gestation. To date, C4d has emerged as the most interesting biomarker of C activation in placenta specimens. This is not surprising since C4d, like C3d, binds covalently to the target cell surface and, being highly stable, acts as a fingerprint of C-mediated activation leading to tissue injury. In conclusion, there are indications to suggest that C is involved in complicated pregnancies, although the precise mechanism by which C is activated is not always clear and remains to be determined.

## Author Contributions

PM and FT designed the study. CC, PL, and LT retrieved the relevant literature. CC and FT drafted the manuscript. PL prepared the figures. PM, LT, and FT revised the literature critically for important intellectual content. All authors approved the final version of the article.

## Conflict of Interest

The authors declare that the research was conducted in the absence of any commercial or financial relationships that could be construed as a potential conflict of interest.

## References

[1] Andreoli L Fredi M Nalli C Reggia R Lojacono A Motta M Pregnancy implications for systemic lupus erythematosus and the antiphospholipid syndrome. *J Autoimmun.* (2012) 38:J197–208. 10.1016/j.jaut.2011.11.010 22204899

[2] Ostensen M Andreoli L Brucato A Cetin I Chambers C Clowse ME State of the art: reproduction and pregnancy in rheumatic diseases. *Autoimmun Rev.* (2015) 14:376–86. 10.1016/j.autrev.2014.12.011 25555818

[3] Meroni PL Borghi MO Raschi E Tedesco F. Pathogenesis of antiphospholipid syndrome: understanding the antibodies. *Nat Rev Rheumatol.* (2011) 7:330–9. 10.1038/nrrheum.2011.52 21556027

[4] Jain V Gordon C. Managing pregnancy in inflammatory rheumatological diseases. *Arthritis Res Ther.* (2011) 13:206. 10.1186/ar3227 21371350 PMC3157639

[5] Andreoli L Bertsias GK Agmon-Levin N Brown S Cervera R Costedoat-Chalumeau N EULAR recommendations for women’s health and the management of family planning, assisted reproduction, pregnancy and menopause in patients with systemic lupus erythematosus and/or antiphospholipid syndrome. *Ann Rheum Dis.* (2017) 76:476–85. 10.1136/annrheumdis-2016-209770 27457513 PMC5446003

[6] Merle NS Noe R Halbwachs-Mecarelli L Fremeaux-Bacchi V Roumenina LT. Complement system part II: role in immunity. *Front Immunol.* (2015) 6:257. 10.3389/fimmu.2015.00257 26074922 PMC4443744

[7] Dunkelberger JR Song WC. Complement and its role in innate and adaptive immune responses. *Cell Res.* (2010) 20:34–50. 10.1038/cr.2009.139 20010915

[8] Freeley S Kemper C Le Friec G. The “ins and outs” of complement-driven immune responses. *Immunol Rev.* (2016) 274:16–32. 10.1111/imr.12472 27782335 PMC5102160

[9] Reis ES Mastellos DC Hajishengallis G Lambris JD. New insights into the immune functions of complement. *Nat Rev Immunol.* (2019) 19:503–16. 10.1038/s41577-019-0168-x 31048789 PMC6667284

[10] Bossi F Fischetti F Pellis V Bulla R Ferrero E Mollnes TE Platelet-activating factor and kinin-dependent vascular leakage as a novel functional activity of the soluble terminal complement complex. *J Immunol.* (2004) 173:6921–7. 10.4049/jimmunol.173.11.6921 15557188

[11] Dobrina A Pausa M Fischetti F Bulla R Vecile E Ferrero E Cytolytically inactive terminal complement complex causes transendothelial migration of polymorphonuclear leukocytes in vitro and in vivo. *Blood.* (2002) 99:185–92. 10.1182/blood.v99.1.185 11756170

[12] Zipfel PF Skerka C. Complement regulators and inhibitory proteins. *Nat Rev Immunol.* (2009) 9:729–40. 10.1038/nri2620 19730437

[13] Bossi F Tripodo C Rizzi L Bulla R Agostinis C Guarnotta C C1q as a unique player in angiogenesis with therapeutic implication in wound healing. *Proc Natl Acad Sci USA.* (2014) 111:4209–14. 10.1073/pnas.1311968111 24591625 PMC3964125

[14] Stevens B Allen NJ Vazquez LE Howell GR Christopherson KS Nouri N The classical complement cascade mediates CNS synapse elimination. *Cell.* (2007) 131:1164–78. 10.1016/j.cell.2007.10.036 18083105

[15] Girardi G Prohaszka Z Bulla R Tedesco F Scherjon S. Complement activation in animal and human pregnancies as a model for immunological recognition. *Mol Immunol.* (2011) 48:1621–30. 10.1016/j.molimm.2011.04.011 21600656

[16] Derzsy Z Prohaszka Z Rigo J Jr. Fust G Molvarec A. Activation of the complement system in normal pregnancy and preeclampsia. *Mol Immunol.* (2010) 47:1500–6. 10.1016/j.molimm.2010.01.021 20181396

[17] Agostinis C Stampalija T Tannetta D Loganes C Vecchi Brumatti L De Seta F Complement component C1q as potential diagnostic but not predictive marker of preeclampsia. *Am J Reprod Immunol.* (2016) 76:475–81. 10.1111/aji.12586 27666323

[18] Jia K Ma L Wu S Yang W. Serum levels of complement factors C1q, Bb, and H in normal pregnancy and severe Pre-Eclampsia. *Med Sci Monit.* (2019) 25:7087–93. 10.12659/MSM.915777 31541546 PMC6767947

[19] Holmes CH Simpson KL Okada H Okada N Wainwright SD Purcell DF Complement regulatory proteins at the feto-maternal interface during human placental development: distribution of CD59 by comparison with membrane cofactor protein (CD46) and decay accelerating factor (CD55). *Eur J Immunol.* (1992) 22:1579–85. 10.1002/eji.1830220635 1376264

[20] Tedesco F Narchi G Radillo O Meri S Ferrone S Betterle C. Susceptibility of human trophoblast to killing by human complement and the role of the complement regulatory proteins. *J Immunol.* (1993) 151:1562–70. 7687635

[21] Bulla R Bossi F Tedesco F. The complement system at the embryo implantation site: friend or foe? *Front Immunol.* (2012) 3:55. 10.3389/fimmu.2012.00055 22566936 PMC3341982

[22] Bulla R Agostinis C Bossi F Rizzi L Debeus A Tripodo C Decidual endothelial cells express surface-bound C1q as a molecular bridge between endovascular trophoblast and decidual endothelium. *Mol Immunol.* (2008) 45:2629–40. 10.1016/j.molimm.2007.12.025 18295334 PMC2632959

[23] Agostinis C Bulla R Tripodo C Gismondi A Stabile H Bossi F An alternative role of C1q in cell migration and tissue remodeling: contribution to trophoblast invasion and placental development. *J Immunol.* (2010) 185:4420–9. 10.4049/jimmunol.0903215 20810993

[24] Girardi G Yarilin D Thurman JM Holers VM Salmon JE. Complement activation induces dysregulation of angiogenic factors and causes fetal rejection and growth restriction. *J Exp Med.* (2006) 203:2165–75. 10.1084/jem.20061022 16923853 PMC2118387

[25] Girardi G. Complement activation, a threat to pregnancy. *Semin Immunopathol.* (2018) 40:103–11. 10.1007/s00281-017-0645-x 28900713

[26] Ohmura K Oku K Kitaori T Amengual O Hisada R Kanda M Pathogenic roles of anti-C1q antibodies in recurrent pregnancy loss. *Clin Immunol.* (2019) 203:37–44. 10.1016/j.clim.2019.04.005 30974291

[27] Sugiura-Ogasawara M Nozawa K Nakanishi T Hattori Y Ozaki Y. Complement as a predictor of further miscarriage in couples with recurrent miscarriages. *Hum Reprod.* (2006) 21:2711–4. 10.1093/humrep/del229 16790609

[28] Micheloud D Sarmiento E Teijeiro R Jensen J Rodriguez Molina JJ Fernandez-Cruz E Hypocomplementemia in the absence of autoantibodies in women with recurrent pregnancy loss. *Allergol Immunopathol*. (2007) 35:90–4. 10.1157/13106775 17594871

[29] Banadakoppa M Chauhan MS Havemann D Balakrishnan M Dominic JS Yallampalli C. Spontaneous abortion is associated with elevated systemic C5a and reduced mRNA of complement inhibitory proteins in placenta. *Clin Exp Immunol.* (2014) 177:743–9. 10.1111/cei.12371 24802103 PMC4137859

[30] Wikstrom AK Larsson A Eriksson UJ Nash P Norden-Lindeberg S Olovsson M. Placental growth factor and soluble FMS-like tyrosine kinase-1 in early-onset and late-onset preeclampsia. *Obstet Gynecol.* (2007) 109:1368–74. 10.1097/01.AOG.0000264552.85436.a1 17540809

[31] Singh J Ahmed A Girardi G. Role of complement component C1q in the onset of preeclampsia in mice. *Hypertension.* (2011) 58:716–24. 10.1161/HYPERTENSIONAHA.111.175919 21859968

[32] Lokki AI Heikkinen-Eloranta J Jarva H Saisto T Lokki ML Laivuori H Complement activation and regulation in preeclamptic placenta. *Front Immunol.* (2014) 5:312. 10.3389/fimmu.2014.00312 25071773 PMC4088925

[33] Than NG Romero R Erez O Kusanovic JP Tarca AL Edwin SS A role for mannose-binding lectin, a component of the innate immune system in pre-eclampsia. *Am J Reprod Immunol.* (2008) 60:333–45. 10.1111/j.1600-0897.2008.00631.x 18727690 PMC2775464

[34] Agostinis C Bossi F Masat E Radillo O Tonon M De Seta F MBL interferes with endovascular trophoblast invasion in pre-eclampsia. *Clin Dev Immunol.* (2012) 2012:484321. 10.1155/2012/484321 22203857 PMC3235499

[35] Regal JF Lund JM Wing CR Root KM McCutcheon L Bemis LT Interactions between the complement and endothelin systems in normal pregnancy and following placental ischemia. *Mol Immunol.* (2019) 114:10–8. 10.1016/j.molimm.2019.06.015 31326653 PMC6774867

[36] Haeger M Bengtson A Karlsson K Heideman M. Complement activation and anaphylatoxin (C3a and C5a) formation in preeclampsia and by amniotic fluid. *Obstet Gynecol.* (1989) 73:551–6. 2784554

[37] Denny KJ Coulthard LG Finnell RH Callaway LK Taylor SM Woodruff TM. Elevated complement factor C5a in maternal and umbilical cord plasma in preeclampsia. *J Reprod Immunol.* (2013) 97:211–6. 10.1016/j.jri.2012.11.006 23415845

[38] Burwick RM Fichorova RN Dawood HY Yamamoto HS Feinberg BB. Urinary excretion of C5b-9 in severe preeclampsia: tipping the balance of complement activation in pregnancy. *Hypertension.* (2013) 62:1040–5. 10.1161/HYPERTENSIONAHA.113.01420 24060886

[39] Buurma A Cohen D Veraar K Schonkeren D Claas FH Bruijn JA Preeclampsia is characterized by placental complement dysregulation. *Hypertension.* (2012) 60:1332–7. 10.1161/HYPERTENSIONAHA.112.194324 23006730

[40] Yonekura Collier AR Zsengeller Z Pernicone E Salahuddin S Khankin EV Karumanchi SA. Placental sFLT1 is associated with complement activation and syncytiotrophoblast damage in preeclampsia. *Hypertens Pregnancy.* (2019) 38:193–9. 10.1080/10641955.2019.1640725 31291799 PMC6707710

[41] Lynch AM Gibbs RS Murphy JR Byers T Neville MC Giclas PC Complement activation fragment Bb in early pregnancy and spontaneous preterm birth. *Am J Obstet Gynecol.* (2008) 199:354.e1–8. 10.1016/j.ajog.2008.07.044 18928972 PMC2586079

[42] Lynch AM Gibbs RS Murphy JR Giclas PC Salmon JE Holers VM. Early elevations of the complement activation fragment C3a and adverse pregnancy outcomes. *Obstet Gynecol.* (2011) 117:75–83. 10.1097/AOG.0b013e3181fc3afa 21173647 PMC5267353

[43] Lappas M Woodruff TM Taylor SM Permezel M. Complement C5A regulates prolabor mediators in human placenta. *Biol Reprod.* (2012) 86:190. 10.1095/biolreprod.111.098475 22441801

[44] de Groot PG Meijers JC. beta(2) -Glycoprotein I: evolution, structure and function. *J Thromb Haemost.* (2011) 9:1275–84. 10.1111/j.1538-7836.2011.04327.x 21535391

[45] McDonnell T Wincup C Buchholz I Pericleous C Giles I Ripoll V The role of beta-2-glycoprotein I in health and disease associating structure with function: more than just APS. *Blood Rev.* (2020) 39:100610. 10.1016/j.blre.2019.100610 31471128 PMC7014586

[46] Agostinis C Biffi S Garrovo C Durigutto P Lorenzon A Bek A In vivo distribution of beta2 glycoprotein I under various pathophysiologic conditions. *Blood.* (2011) 118:4231–8. 10.1182/blood-2011-01-333617 21791419

[47] Chighizola CB Andreoli L Gerosa M Tincani A Ruffatti A Meroni PL. The treatment of anti-phospholipid syndrome: A comprehensive clinical approach. *J Autoimmun.* (2018) 90:1–27. 10.1016/j.jaut.2018.02.003 29449131

[48] Tabacco S Giannini A Garufi C Botta A Salvi S Del Sordo G Complementemia in pregnancies with antiphospholipid syndrome. *Lupus.* (2019) 28:1503–9. 10.1177/0961203319882507 31623520

[49] Reggia R Ziglioli T Andreoli L Bellisai F Iuliano A Gerosa M Primary anti-phospholipid syndrome: any role for serum complement levels in predicting pregnancy complications? *Rheumatology*. (2012) 51:2186–90. 10.1093/rheumatology/kes225 22923750

[50] Ruffatti A Tonello M Visentin MS Bontadi A Hoxha A De Carolis S Risk factors for pregnancy failure in patients with anti-phospholipid syndrome treated with conventional therapies: a multicentre, case-control study. *Rheumatology*. (2011) 50:1684–9. 10.1093/rheumatology/ker139 21652586

[51] De Carolis S Botta A Santucci S Salvi S Moresi S Di Pasquo E Complementemia and obstetric outcome in pregnancy with antiphospholipid syndrome. *Lupus.* (2012) 21:776–8. 10.1177/0961203312444172 22635230

[52] Deguchi M Yamada H Sugiura-Ogasawara M Morikawa M Fujita D Miki A Factors associated with adverse pregnancy outcomes in women with antiphospholipid syndrome: a multicenter study. *J Reprod Immunol.* (2017) 122:21–7. 10.1016/j.jri.2017.08.001 28837832

[53] Kim MY Guerra MM Kaplowitz E Laskin CA Petri M Branch DW Complement activation predicts adverse pregnancy outcome in patients with systemic lupus erythematosus and/or antiphospholipid antibodies. *Ann Rheum Dis.* (2018) 77:549–55. 10.1136/annrheumdis-2017-212224 29371202 PMC6037302

[54] Scambi C Ugolini S Tonello M Bortolami O De Franceschi L Castagna A Complement activation in the plasma and placentas of women with different subsets of antiphospholipid syndrome. *Am J Reprod Immunol.* (2019) 82:e13185. 10.1111/aji.13185 31479579

[55] Cohen D Buurma A Goemaere NN Girardi G le Cessie S Scherjon S Classical complement activation as a footprint for murine and human antiphospholipid antibody-induced fetal loss. *J Pathol.* (2011) 225:502–11. 10.1002/path.2893 21688269

[56] Matrai CE Rand JH Baergen RN. Absence of distinct immunohistochemical distribution of annexin A5, C3b, C4d, and C5b-9 in placentas from patients with antiphospholipid antibodies, preeclampsia, and systemic lupus erythematosus. *Pediatr Dev Pathol.* (2019) 22:431–9. 10.1177/1093526619836025 30922166

[57] Shamonki JM Salmon JE Hyjek E Baergen RN. Excessive complement activation is associated with placental injury in patients with antiphospholipid antibodies. *Am J Obstet Gynecol.* (2007) 196:167.e1–167.e5. 10.1016/j.ajog.2006.10.879 17306667 PMC2248299

[58] Tedesco F Borghi MO Gerosa M Chighizola CB Macor P Lonati PA Pathogenic role of complement in antiphospholipid syndrome and therapeutic implications. *Front Immunol.* (2018) 9:1388. 10.3389/fimmu.2018.01388 29971066 PMC6018396

[59] Holers VM Girardi G Mo L Guthridge JM Molina H Pierangeli SS Complement C3 activation is required for antiphospholipid antibody-induced fetal loss. *J Exp Med.* (2002) 195:211–20. 10.1084/jem.200116116 11805148 PMC2193604

[60] Girardi G Berman J Redecha P Spruce L Thurman JM Kraus D Complement C5a receptors and neutrophils mediate fetal injury in the antiphospholipid syndrome. *J Clin Invest.* (2003) 112:1644–54. 10.1172/JCI18817 14660741 PMC281643

[61] Thurman JM Kraus DM Girardi G Hourcade D Kang HJ Royer PA A novel inhibitor of the alternative complement pathway prevents antiphospholipid antibody-induced pregnancy loss in mice. *Mol Immunol.* (2005) 42:87–97. 10.1016/j.molimm.2004.07.043 15488947

[62] Berman J Girardi G Salmon JE. TNF-alpha is a critical effector and a target for therapy in antiphospholipid antibody-induced pregnancy loss. *J Immunol.* (2005) 174:485–90. 10.4049/jimmunol.174.1.485 15611274

[63] Salmon JE Girardi G. Antiphospholipid antibodies and pregnancy loss: a disorder of inflammation. *J Reprod Immunol.* (2008) 77:51–6. 10.1016/j.jri.2007.02.007 17418423 PMC2247372

[64] Meroni PL Macor P Durigutto P De Maso L Gerosa M Ferraresso M Complement activation in antiphospholipid syndrome and its inhibition to prevent rethrombosis after arterial surgery. *Blood.* (2016) 127:365–7. 10.1182/blood-2015-09-672139 26644452

[65] Gustavsen A Skattum L Bergseth G Lorentzen B Floisand Y Bosnes V Effect on mother and child of eculizumab given before caesarean section in a patient with severe antiphospholipid syndrome: a case report. *Medicine*. (2017) 96:e6338. 10.1097/MD.0000000000006338 28296762 PMC5369917

[66] Agostinis C Durigutto P Sblattero D Borghi MO Grossi C Guida F A non-complement-fixing antibody to beta2 glycoprotein I as a novel therapy for antiphospholipid syndrome. *Blood.* (2014) 123:3478–87. 10.1182/blood-2013-11-537704 24642748

[67] Tsokos GC Lo MS Costa Reis P Sullivan KE. New insights into the immunopathogenesis of systemic lupus erythematosus. *Nat Rev Rheumatol.* (2016) 12:716–30. 10.1038/nrrheum.2016.186 27872476

[68] Siegert C Daha M Westedt ML van der Voort E Breedveld F. IgG autoantibodies against C1q are correlated with nephritis, hypocomplementemia, and dsDNA antibodies in systemic lupus erythematosus. *J Rheumatol.* (1991) 18:230–4. 2023216

[69] Orbai AM Truedsson L Sturfelt G Nived O Fang H Alarcon GS Anti-C1q antibodies in systemic lupus erythematosus. *Lupus.* (2015) 24:42–9. 10.1177/0961203314547791 25124676 PMC4268323

[70] Biesecker G Katz S Koffler D. Renal localization of the membrane attack complex in systemic lupus erythematosus nephritis. *J Exp Med.* (1981) 154:1779–94. 10.1084/jem.154.6.1779 7033435 PMC2186538

[71] Pickering MC Ismajli M Condon MB McKenna N Hall AE Lightstone L Eculizumab as rescue therapy in severe resistant lupus nephritis. *Rheumatology*. (2015) 54:2286–8. 10.1093/rheumatology/kev307 26316577 PMC4643725

[72] Coppo R Peruzzi L Amore A Martino S Vergano L Lastauka I Dramatic effects of eculizumab in a child with diffuse proliferative lupus nephritis resistant to conventional therapy. *Pediatr Nephrol.* (2015) 30:167–72. 10.1007/s00467-014-2944-y 25173358

[73] Bao L Cunningham PN Quigg RJ. Complement in lupus nephritis: new perspectives. *Kidney Dis*. (2015) 1:91–9. 10.1159/000431278 27536669 PMC4934819

[74] Wang Y Hu Q Madri JA Rollins SA Chodera A Matis LA. Amelioration of lupus-like autoimmune disease in NZB/WF1 mice after treatment with a blocking monoclonal antibody specific for complement component C5. *Proc Natl Acad Sci USA.* (1996) 93:8563–8. 10.1073/pnas.93.16.8563 8710910 PMC38712

[75] Trouw LA Pickering MC Blom AM. The complement system as a potential therapeutic target in rheumatic disease. *Nat Rev Rheumatol.* (2017) 13:538–47. 10.1038/nrrheum.2017.125 28794515

[76] Macedo AC Isaac L. Systemic lupus erythematosus and deficiencies of early components of the complement classical pathway. *Front Immunol.* (2016) 7:55. 10.3389/fimmu.2016.00055 26941740 PMC4764694

[77] Swaak AJ Groenwold J Bronsveld W. Predictive value of complement profiles and anti-dsDNA in systemic lupus erythematosus. *Ann Rheum Dis.* (1986) 45:359–66. 10.1136/ard.45.5.359 3487292 PMC1001892

[78] Sandhu V Quan M. SLE and serum complement: causative, concomitant or coincidental? *Open Rheumatol J.* (2017) 11:113–22. 10.2174/1874312901711010113 29290848 PMC5737025

[79] Aringer M Costenbader K Daikh D Brinks R Mosca M Ramsey-Goldman R 2019 European league against rheumatism/american college of rheumatology classification criteria for systemic lupus erythematosus. *Ann Rheum Dis.* (2019) 78:1151–9. 10.1136/annrheumdis-2018-214819 31383717

[80] Batal I Liang K Bastacky S Kiss LP McHale T Wilson NL Prospective assessment of C4d deposits on circulating cells and renal tissues in lupus nephritis: a pilot study. *Lupus.* (2012) 21:13–26. 10.1177/0961203311422093 21959138

[81] Putterman C Furie R Ramsey-Goldman R Askanase A Buyon J Kalunian K Cell-bound complement activation products in systemic lupus erythematosus: comparison with anti-double-stranded DNA and standard complement measurements. *Lupus Sci Med.* (2014) 1:e000056. 10.1136/lupus-2014-000056 25396070 PMC4225732

[82] Mehta B Luo Y Xu J Sammaritano L Salmon J Lockshin M Trends in maternal and fetal outcomes among pregnant women with systemic lupus erythematosus in the United States: a cross-sectional analysis. *Ann Intern Med.* (2019) 171:164–71. 10.7326/M19-0120 31284305

[83] Skorpen CG Lydersen S Gilboe IM Skomsvoll JF Salvesen KA Palm O Influence of disease activity and medications on offspring birth weight, pre-eclampsia and preterm birth in systemic lupus erythematosus: a population-based study. *Ann Rheum Dis.* (2018) 77:264–9. 10.1136/annrheumdis-2017-211641 29092851

[84] Buyon JP Kim MY Guerra MM Lu S Reeves E Petri M Kidney outcomes and risk factors for nephritis (Flare/De Novo) in a multiethnic cohort of pregnant patients with lupus. *Clin J Am Soc Nephrol.* (2017) 12:940–6. 10.2215/CJN.11431116 28400421 PMC5460714

[85] Mok CC. Epidemiology and survival of systemic lupus erythematosus in Hong Kong Chinese. *Lupus.* (2011) 20:767–71. 10.1177/0961203310388447 21148605

[86] Al Arfaj AS Khalil N. Pregnancy outcome in 396 pregnancies in patients with SLE in Saudi Arabia. *Lupus.* (2010) 19:1665–73. 10.1177/0961203310378669 20947541

[87] Buyon JP Kim MY Guerra MM Laskin CA Petri M Lockshin MD Predictors of pregnancy outcomes in patients with lupus: a cohort study. *Ann Intern Med.* (2015) 163:153–63. 10.7326/M14-2235 26098843 PMC5113288

[88] Clowse ME Wallace DJ Weisman M James A Criscione-Schreiber LG Pisetsky DS. Predictors of preterm birth in patients with mild systemic lupus erythematosus. *Ann Rheum Dis.* (2013) 72:1536–9. 10.1136/annrheumdis-2012-202449 23361085

[89] Abramson SB Buyon JP. Activation of the complement pathway: comparison of normal pregnancy, preeclampsia, and systemic lupus erythematosus during pregnancy. *Am J Reprod Immunol.* (1992) 28:183–7. 10.1111/j.1600-0897.1992.tb00787.x 1285875

[90] Buyon JP Tamerius J Ordorica S Young B Abramson SB. Activation of the alternative complement pathway accompanies disease flares in systemic lupus erythematosus during pregnancy. *Arthritis Rheum.* (1992) 35:55–61. 10.1002/art.1780350109 1731815

[91] Minamiguchi S Mikami Y Nakajima N Salah A Kondoh E Tatsumi K Complement split product C4d deposition in placenta in systemic lupus erythematosus and pregnancy-induced hypertension. *Pathol Int.* (2013) 63:150–7. 10.1111/pin.12041 23530559

[92] Lonati PA Scavone M Gerosa M Borghi MO Pregnolato F Curreli D Blood cell-bound C4d as a marker of complement activation in patients with the antiphospholipid syndrome. *Front Immunol.* (2019) 10:773. 10.3389/fimmu.2019.00773 31031764 PMC6474283

[93] Girardi G Redecha P Salmon JE. Heparin prevents antiphospholipid antibody-induced fetal loss by inhibiting complement activation. *Nat Med.* (2004) 10:1222–6. 10.1038/nm1121 15489858

[94] Mok CC Wong RW. Pregnancy in systemic lupus erythematosus. *Postgrad Med J.* (2001) 77:157–65. 10.1136/pmj.77.905.157 11222822 PMC1741938

